# Semaphorin3A–neuropilin1 signalling is involved in the generation of cortical interneurons

**DOI:** 10.1007/s00429-016-1337-3

**Published:** 2016-11-17

**Authors:** William D. Andrews, Melissa Barber, Marion Nemitz, Fani Memi, John G. Parnavelas

**Affiliations:** Department of Cell and Developmental Biology, University College London, Gower Street, London, WC1E 6BT UK

**Keywords:** Proliferation, Neuronal migration, Semaphorin, Neuropilin, Interneurons

## Abstract

Cortical interneurons are generated predominantly in the medial ganglionic eminence of the ventral telencephalon and migrate to the cortex during embryonic development. These cells express neuropilin (Nrp1 and Nrp2) receptors which mediate their response to the chemorepulsive class 3 semaphorin (Sema) ligands. We show here that semaphorins Sema3A and Sema3F are expressed in layers adjacent to cortical interneuron migratory streams as well as in the striatum, suggesting they may have a role in guiding these cells throughout their journey. Analysis of *Sema3A* ^−*/*−^ and *Sema3F* ^−*/*−^ mice during corticogenesis showed that absence of Sema3A, but not Sema3F, leads to aberrant migration of cortical interneurons through the striatum. Reduced number of cortical interneurons was found in the cortex of *Sema3A* ^−*/*−^, *Nrp1* ^−*/*−^ and *Nrp2* ^−*/*−^ mice, as well as altered distribution in *Sema3F* ^−*/*−^, *Nrp1* ^−*/*−^, *Nrp2* ^−*/*−^ animals and especially in neuropilin double mutants. The observed decrease in interneurons in *Sema3A* ^−*/*−^ and *Nrp1* ^−*/*−^ mice was due to altered proliferative activity of their progenitors highlighted by changes in their mitotic spindle positioning and angle of cleavage plane during cell division. These findings point to a novel role for Sema3A–Nrp1 signalling in progenitor cell dynamics and in the generation of interneurons in the ventral telencephalon.

**Electronic supplementary material:**

The online version of this article (doi:10.1007/s00429-016-1337-3) contains supplementary material, which is available to authorized users.

## Introduction

The vast majority of cortical interneurons are generated in the medial ganglionic eminence (MGE) of the subpallium and travel tortuous routes to reach their positions in the developing cortical plate (CP) (Hansen et al. 2013; Lavdas et al. 1999; Wichterle et al. 2001). Along their journey, they use an array of guidance cues for positional information, and these include the class 3 semaphorin ligands and their corresponding neuropilin (Nrp) and plexin receptors (Hernandez-Miranda et al. 2011; Marin et al. 2001; Tamamaki et al. 2003a). Several studies have shown that blocking Nrp1 function with either Nrp1-dominant negative construct in slice cultures (Marin et al. 2001) or with anti-Nrp1 antibody (Tamamaki et al. 2003a) leads to interneurons ectopically entering the striatum. Similarly, loss of Nrp2 function in *Nrp2* ^−*/*−^ genetically modified mice shows more cortical and striatal (NPY-expressing) interneurons in the striatum (Marin et al. 2001). Further, in utero electroporation of mouse embryos with small interfering RNAs (siRNAs) to PlexinA1 or its downstream target Limk2 in interneurons results in their aberrant invasion of the developing striatum (Andrews et al. 2013).

Ectopic expression studies of Sema3A and Sema3F at the cortical/striatal boundary in slice cultures have shown a small reduction in the number of interneurons entering the cortex when used alone, but the reduction was greater when used together (Marin et al. 2001; Tamamaki et al. 2003a). Interestingly, the direction of cell migration could be reversed by placing Sema3A- and Sema3F-co-expressing COS-7 cell clusters at the distal end of neocortical slices, but only half the effect was observed when either Sema3A or Sema3F was used alone, again suggesting more than one receptor was involved in semaphorin signalling (Tamamaki et al. 2003a). These findings indicate that semaphorins acting via Nrp/plexin receptors are required for sorting the migrating cortical and striatal interneurons to their correct destinations.

To further explore the role of semaphorins and their receptors in interneuron migration and development, here we analysed the interneuron phenotypes of *Sema3A* ^−/−^ and *Sema3F* ^−*/*−^ mice in relation to those of *Nrp1* ^−*/*−^ animals lacking the semaphorin binding domain, and to *Nrp2* ^−*/*−^ and double neuropilin (*Nrp1* ^−*/*−^ *;Nrp2* ^−*/*−^) knockout mice. Our results point to the importance of Sema3A and Nrp1, and to a lesser extent Sema3F and Nrp2, in the generation and migration of interneurons in the mammalian forebrain. Specifically, the analyses showed that loss of Sema3A or Nrp1 function in interneuron progenitor cells in the MGE resulted in reduced proliferation, likely as a consequence of malformation of the mitotic spindle assembly and changes in the angle of cleavage plane during division. The resemblance of interneuron defects found in *Nrp1* and *Sema3A* mutants strongly suggests that the former mediates the function of Sema3A, and that Sema3F plays a largely redundant role. Our findings point to a novel role for Sem3A–Nrp1 signalling in the genesis and migration of cortical interneurons.

## Materials and methods

### Animals

All experimental procedures were performed in accordance with the UK Animals (Scientific Procedures) Act 1986 and institutional guidelines. Wild-type animals were C57/bl6 J mice obtained from Charles River Ltd. *Nrp1* ^−*/*−^, *Nrp2* ^−*/*−^, *Sema3A* ^−*/*−^ and *Sema3F* ^−*/*−^ mice were generated as described previously (Giger et al. 2000; Gu et al. 2003; Sahay et al. 2003; Taniguchi et al. 1997). *GAD67*-*GFP* ^*neo/*−^ mice (Tamamaki et al. 2003b) were also maintained in C57/Bbl6J background. The day the vaginal plug was found was considered as embryonic day (*E*) 0.5. Animals of both sexes were used in our experiments.

### In situ hybridization

For in situ hybridization and immunohistochemistry, embryonic brains were dissected in phosphate buffered saline (PBS) and fixed in PBS containing 4% paraformaldehyde (PFA) for 4–8 h at room temperature (RT). Following fixation, embryonic brains were cryoprotected in 30% sucrose in diethyl pyrocarbonate (DEPC)-treated PBS, embedded and frozen in a mixture of 15% sucrose/50% Tissue-Tek OCT (Sakura Finetek), and sectioned in the coronal plane at 20 µm using a Cryostat (Bright Instruments). Sections were dried at RT for 2 h before overnight incubation at 65 °C in hybridization buffer [a DEPC-treated solution containing 200 mM NaCl, 5 mM EDTA, 10 mM Tris pH 7.5, 5 mM NaH_2_PO_4_.2H_2_O, 5 mM Na_2_HPO4 (Merck KGaA); 50% deionized formamide (Ambion); 0.1 mg/ml RNAse-free yeast tRNA (Thermo-Fisher Scientific); 1XRNase/DNase-free Denhardts (Thermo-Fisher Scientific); 10% dextran-sulphate (Merck KGaA)] containing 100-500 ng/ml DIG-labelled RNA probes. Probes used were: Sema3A *and Sema3F* (kindly provided by Professor Christiana Ruhrberg, UCL, UK) and glutamic acid decarboxylase 67 (*GAD67*, kindly provided by Dr. Brian Condie, University of Georgia, USA). Following hybridization, sections were washed three times in 50% formamide 1X SSC (Ambion) and 0.1% Tween-20 (Merck KGaA) at 65 °C and two times at RT in 1X MABT (20 mM maleic acid, 30 mM NaCl, 0.1% Tween-20; Merck KGaA) before incubating in blocking solution [MABT containing 2% of blocking reagent (Roche) and 10% of normal goat serum (Vector Laboratories)] followed by overnight incubation in alkaline phosphatase-conjugated anti-DIG antibody (1:1500; Roche). Nitroblue tetrazolium chloride/5-bromo-4-chloro-3-indolyl phosphate (Roche) diluted 1:1000 in MABT containing 5% polyvinyl alcohol (VWR International) was used for the colorimetric detection and Fast Red (Roche) dissolved in 100 mM Tris (pH 8.0) and 400 mM NaCl for fluorescent colour detection by incubation at 37 °C. Fluorescence in situ hybridization was followed by immuno-histochemical detection of Green Fluorescent Protein (GFP) as described below. Sections were mounted with Glycergel Mounting Medium (Dako).

### Immunohistochemistry

Embryonic brain sections were washed in PBS, blocked in a solution of 5% normal goat serum (Merck KGaA) (v/v) containing 0.1% Triton X-100 (v/v) (Merck KGaA) in PBS at RT for 1 h. They were subsequently incubated in primary antibodies at RT for 2 h and, then, at 4 °C overnight. The following antibodies were used: mouse monoclonal 5-Bromodeoxyuridine (BrdU; 1:1000; Progen), acetylated Tubulin (6-1 B-1; 1:200; Millipore), rabbit polyclonal raised against calbindin (CB-28; 1:3000; Swant), cleaved caspase-3 (CC3; 1:250; Cell Signaling Technology), Forkhead box protein P2 (FOXP2) (ab16046; 1:700; Abcam), Phosphohistone H-3 (PH-3; 1:1000; Millipore), atypical protein kinase C (aPKC, C-20; 1:200; Santa Cruz) and chicken polyclonal raised against GFP (1:500; Aves Laboratories). Following incubation in primary antibodies, sections were washed in PBS, incubated in biotinylated anti-species (1:250; Vector Laboratories) for 2 h, and processed using conventional immunohistochemistry protocols described previously (Andrews et al. 2008).

### Interneuron counts in the cortex

Counts of interneurons labelled with *GAD67* or CB were made from images collected with a Leica Microsystems light microscope. The images were of coronal strips (300 μm wide) spanning the thickness of the neocortex throughout its rostro-caudal extent at different ages (minimum of six sections at each level (rostral, middle, caudal) from each of three animals for each genotype). In all counts, the experimenter did not know the genotype of the animals. Each coronal strip was divided into bins arranged parallel to the pial surface that corresponded to the different layers of the developing cortex.

### Quantification of labelled cells in the striatum

All morphometric analyses were conducted separately for the rostral, middle and caudal levels of the striatum based on the following anatomical landmarks. The rostral level was considered where the septum was clearly identifiable, the middle level was selected where the intraventricular foramen and the anterior-dorsal thalamus were present, and the caudal level was chosen where the telo-diencephalic junction was distinguishable and the caudal ganglionic eminence was present.

To determine striatal area, sections were stained with 0.025% thionin solution for 2 min and rinsed through an ascending series of alcohols (70–100%). Striatal area was estimated using Image J software (ImageJ; NIH, version 1.48). To assess the total number of immunoreactive cells throughout the rostral-caudal extend of the striatum, a minimum of three non-consecutive sections were stained for each marker per animal, age and genotype.

### Dissociated MGE cell cultures

Dissociated cell cultures were prepared from embryonic mice as described previously (Cavanagh et al. 1997). Briefly, MGEs were dissected out in cold artificial cerebrospinal fluid (ACSF) under a stereomicroscope. They were incubated in neurobasal medium (Thermo-Fisher Scientific) containing 0.05% trypsin (Merck KGaA) and 100 µg/ml DNaseI (Roche) at 37 °C for 15 min. Trypsinization was quenched with neurobasal medium containing 10% of FBS (Thermo-Fisher Scientific) at 37 °C for 5 min. MGEs were then triturated by pipetting until no cellular aggregates were visible. The homogenous cell suspensions were subsequently pelleted by centrifugation at 1000×*g* for 3 min. Cells were re-suspended in dissociation media (DM) [DMEM/F12 culture media containing B27 supplement, 100 µg/ml penicillin/streptomycin and 2 mM l-glutamine (Thermo-Fisher Scientific)], and 100,000 cells were seeded onto 13 mm coverslips coated previously with 10 µg/ml poly-l-lysine and 10 µg/ml laminin (Merck KGaA), and incubated in a humidified incubator at 37 °C.

### Quantification of neuropilin expression

For quantification of Nrp receptor expression, 24 h after plating, E13.5 MGE cells were fixed in 1% PFA on ice for 10 min. Cells were washed and blocked in a solution of 5% normal goat serum (Merck KGaA) (v/v) containing 0.1% Triton X-100 (v/v) (Merck KGaA) in PBS at RT for 2 h and, then, incubated in blocking solution containing goat anti-Nrp1-fluorescein conjugated (1 µg/ml; R&D Systems) and mouse monoclonal anti-Nrp2-APC conjugated antibodies (1 µg/ml; R&D Systems) at RT for 2 h. They were subsequently washed, post-fixed and their nuclei counterstained with DAPI (2.5 μg/ml; Merck KGaA). Six images were taken per coverslip in triplicate (40X magnification), and labelled cells were counted.

### Proliferation

The mothers of E14.5 embryos were injected intraperitoneally with 50 μg of BrdU (Merck KGaA) per gram of body weight and killed 1 h later. Brains were then fixed with 4% PFA, and 20 μm-thick sections were cut with a Cryostat. For BrdU immunolabelling, sections were first incubated in 2 N HCl at 37 °C for 30 min to unmask the antigen, followed by three washes in PBS. For PH-3 staining, the remaining of the procedure was performed as described above. We counted all PH-3+ cells in 200 µm wide strips perpendicular to the ventricular wall of the VZ, and in a 4 × 10^4^ µm^2^ area within the subventricular zone (SVZ) of the MGE. Apical progenitors are defined here as PH3+ cells within three cell diameters from the ventricular surface, and basal progenitors are those that are more than three cells away.

### Angle measurements

Coronal sections taken from E12.5 embryonic brains were stained with the apical marker aPKC and nuclei counterstained with DAPI. Cell division angle measurements from the horizontal plane of nuclei at anaphase in the VZ of the MGE were measured using ImageJ 1.48 software.

### Digital image acquisition and processing

Optical and fluorescent images were collected using Leica Microsystems light and confocal microscopes. Images were reconstructed and digitized with Photoshop CS2 software (Adobe Systems).

### Statistics

All data are reported as mean number and SEM. Statistical analysis was performed with Graph-Pad 3 (Graph-Pad Software). A one-way ANOVA was used to evaluate the fit of the data to a normal distribution and, then, Student’s *t* test was used to evaluate paired comparisons. Significant differences were considered when *p* < 0.05.

## Results

### Expression of Sema3A and Sema3F along interneuron migratory routes

**Fig. 1:**
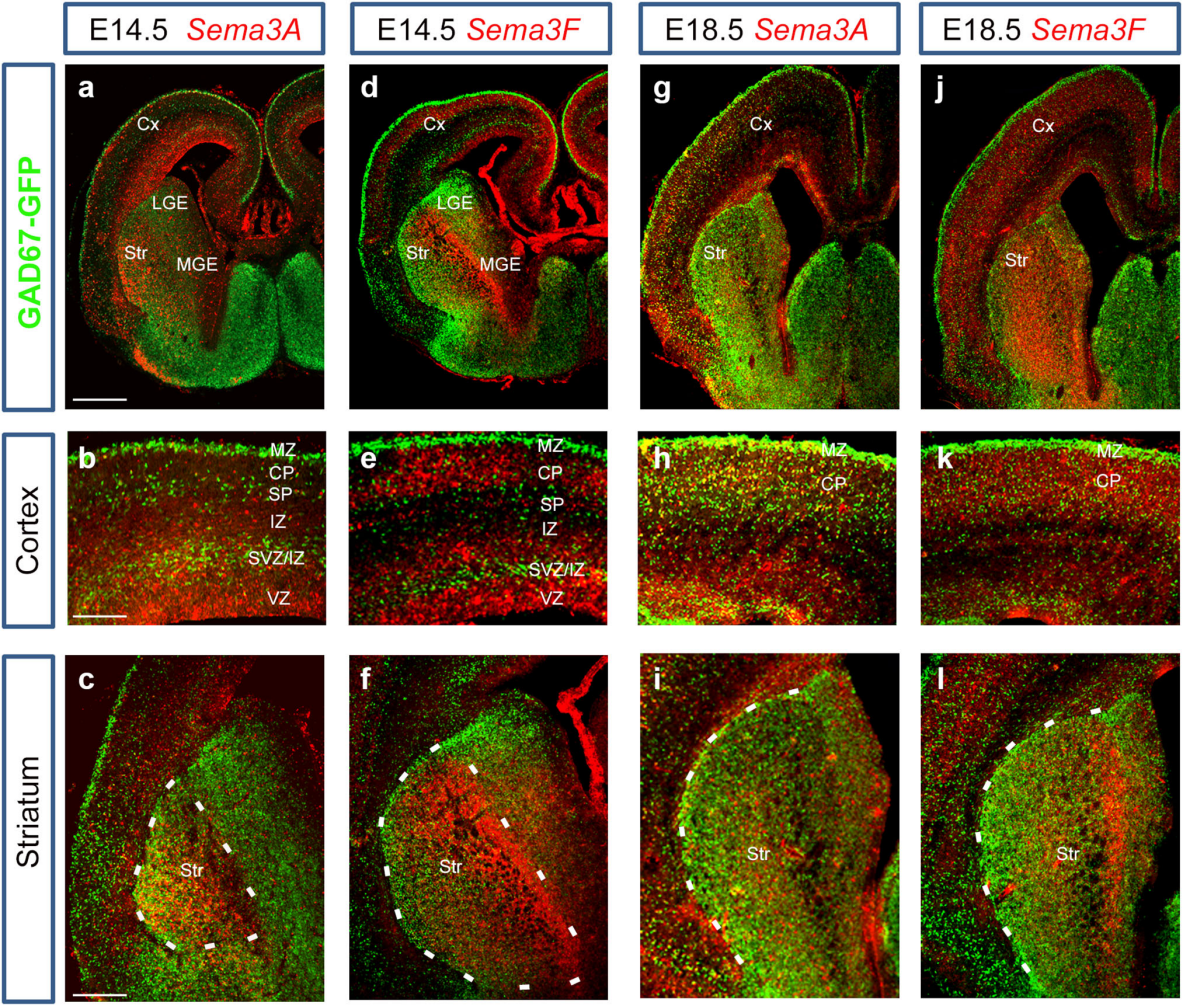
Expression patterns of *Sema3A* and *Sema3F* in GAD67-GFP mouse brains. *In situ* hybridization for *Sema3A* (**a**–**c**, **g**–**i**) and *Sema3F* (**d**–**f**, **j**–**l**) in coronal sections at E14.5 (**a**–**f**) and E18.5 (**g**–**l**). Complementary expression patterns were observed between *Sema3A/Sema3F* and GAD67-GFP in the cortex and striatum at both ages.* Scale bars* **a**, 500 µm; **b**, 150 µm; **c**, 300 µm. *CP* cortical plate, *Cx* cerebral cortex, *IZ* intermediate zone, *LV* lateral ventricle, *MGE* medial ganglionic eminence, *SP* subplate, *Str* striatum, *SVZ* subventricular zone, *VZ* ventricular zone

Previous studies have shown a role for Nrp receptors in interneuron development, but no clear function for their chemorepulsive ligands, Sema3A and Sema3F. To address this, we first compared the expression patterns of *Sema3A* and *Sema3F* and the cortical interneuron marker GAD67 (Retaux et al. 1993) in GAD67-GFP transgenic mice (Tamamaki et al. 2003b) at middle (E14.5) and late (E18.5) stages of corticogenesis. At E14.5, the expression patterns of *Sema3A* and *Sema3F* in the dorsal cortex complemented that of GAD67-GFP, with strong presence of *Sema3A* in the intermediate zone (IZ), of *Sema3F* in the CP, and of both semaphorins in the VZ/SVZ. Thus, Sema3A and Sema3F appear to be expressed abundantly in layers adjacent to interneuron migratory streams (Fig. 1a, b, d, e). In the ventral forebrain, strong expression of both semaphorins was observed in the developing striatum, particularly of *Sema3F*, in agreement with previous studies (Marin et al. 2001; Tamamaki et al. 2003a) (Fig. 1a, c, d, f). At E18.5, expression of both *Sema3A* and *Sema3F* showed similar patterns to E14.5, but at reduced levels in both dorsal and ventral forebrains (Fig. 1g–l). Thus, it appears from the expression patterns that both semaphorins are in positions to guide migrating interneurons through the dorsal and ventral telencephalon.

### Deletion of semaphorins leads to alterations of interneuron numbers

**Fig. 2:**
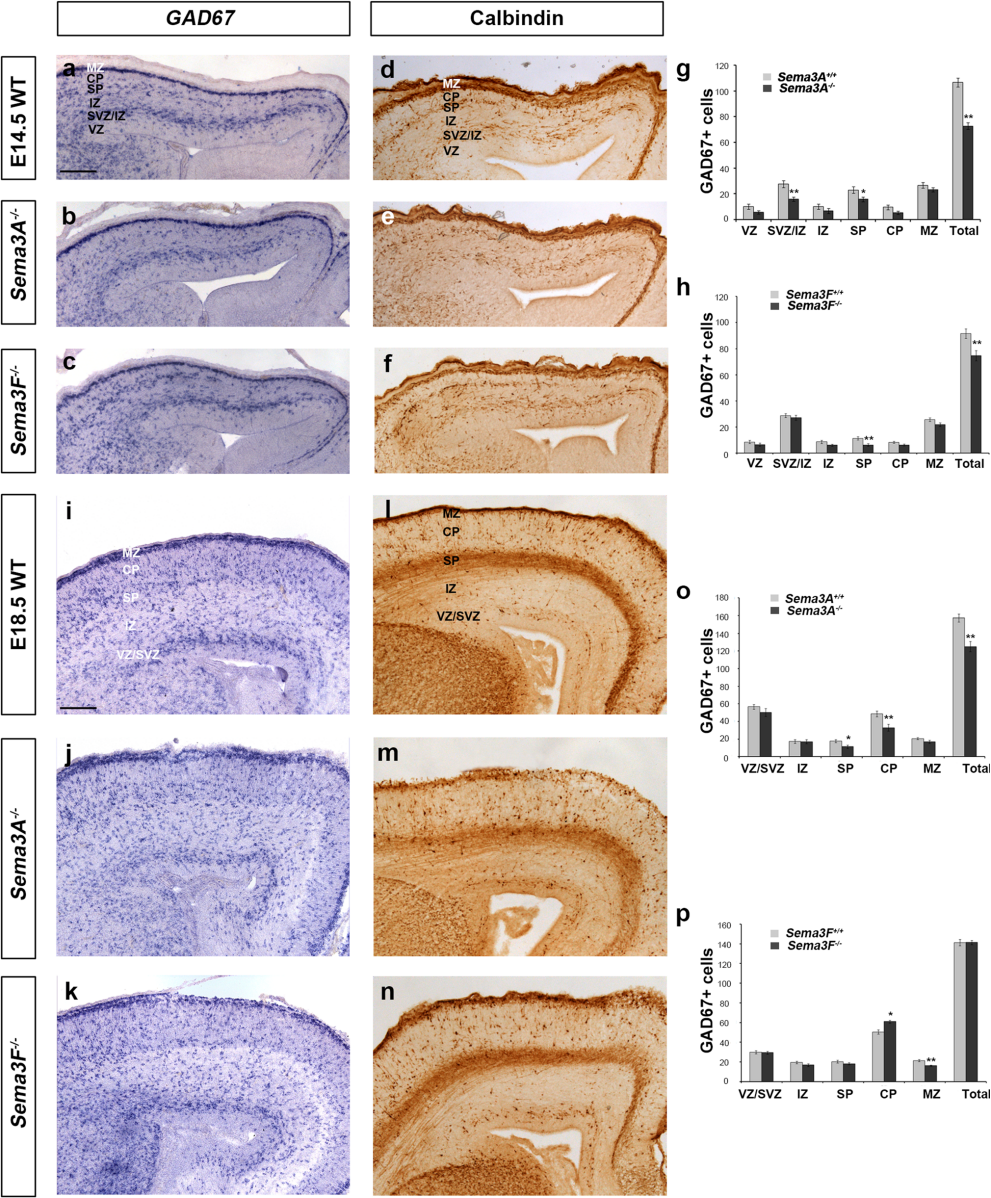
Altered number and distribution of GABAergic interneurons in the cerebral cortex of semaphorin knockout mice. Images of in situ hybridization for *GAD67* (**a**–**c**, **i**–**k**) and immunohistochemistry for CB (**d**–**f**, **l**–**n**) in coronal sections through the cortex of wild-type (**a**, **d**, **i**, **l**), *Sema3A* ^−*/*−^ (**b**, **e**, **j**, **m**) and *Sema3F* ^−*/*−^ (**c**, **f**, **k**, **n**) mice at E14.5 (**a**–**f**) and E18.5 (**i**–**n**). Analysis of the number and distribution of *GAD67* labelled cells in all layers of the cortex of *Sema3A* ^−*/*−^ (**g,o**) and *Sema3F* ^−*/*−^ (**h**, **p**) mice at E14.5 (**g**, **h**) and E18.5 (**o**, **p**). Counts were made in the middle region along the rostro-caudal extent of the cortex. *Scale bar* in **a**, **i** is 100 µm. *Error bars* indicate SEM (Student’s *t* test, *p < 0.01, **p < 0.001). *CP* cortical plate, *IZ* intermediate zone, *MZ* marginal zone, *SP* subplate, *SVZ* subventricular zone, *VZ* ventricular zone

Using in situ hybridization for *GAD67,* we assessed the number of interneurons within the developing cortex in *Sema3A* ^−*/*−^, *Sema3F* ^−*/*−^ and control littermates (*n* = 3 all groups) at middle (E14.5) and late (E18.5) stages. We observed a significant decrease in the total number of *GAD67*+ cells in the cortices of both *Sema3A* ^−*/*−^ (*Sema3A* ^+*/*+^ 106.78 ± 3.51; *Sema3A* ^−*/*−^ 72.8 ± 2.73, *p* < 0.0006) and *Sema3F* ^−*/*−^ mice (*Sema3F* ^+*/*+^ 91.76 ± 3.65; *Sema3F* ^−*/*−^ 74.84.46 ± 3.92, *p* < 0.001) compared to control littermates (Fig. 2a–c, g, h) at E14.5. These observations were confirmed using immunohistochemistry for the interneuron marker CB (Anderson et al. 1997) (Fig. 2d–f). To assess potential changes in the laminar distribution of these neurons, we quantified the presence of GAD67+ cells in each cortical layer as a percentage of the total for each genotype. This analysis showed that loss of Sema3A signalling in the cortex leads to a significant increase in the number of GAD67+ cells in the MZ (*Sema3A* ^+*/*+^ 24.91 ± 2.05%; *Sema3A* ^−*/*−^ 31.96 ± 3.75%, *p* < 0.04), and a concomitant decrease in the VZ, CP and SVZ/IZ (Supplementary Fig. 1a). In *Sema3F* ^−*/*−^ mice of the same age, we observed a small, but significant shift of GAD67+ cells from the SP (*Sema3F* ^+*/*+^ 12.44 ± 1.32%; *Sema3F* ^−*/*−^ 8.57 ± 1.27%, *p* < 0.03) into the SVZ/IZ (*Sema3F* ^+*/*+^ 31.46 ± 1.71%; *Sema3F* ^−*/*−^ 36.53 ± 2.58%, *p* < 0.02) (Supplementary Fig. 1b).

Analysis at a later phase of corticogenesis (E18.5) revealed a significant decrease in the total number of GAD67+ cells in the middle regions (along the rostral-caudal axis) of the cortex of *Sema3A* ^−*/*−^ mice (*Sema3A* ^+*/*+^ 157.41 ± 4.56; *Sema3A* ^−*/*−^ 125.09 ± 5.94, *p* < 0.008) compared to control littermates, especially in the SP and CP (Fig. 2i, j, o), but no marked differences in the cortex of *Sema3F* ^−*/*−^ (*Sema3F* ^+*/*+^ 141.33 ± 3.36; *Sema3F* ^−*/*−^ 141.52 ± 2.14, *p* < 0.956) animals compared to controls (Fig. 2i, k, p). These results were again confirmed using CB immunohistochemistry (Fig. *2*l–n). When we assessed the laminar distribution of GAD6+ cells in the cortex of these mice, we found no changes in *Sema3A* ^−*/*−^ nulls (Supplementary Fig. 1c), but we did in *Sema3F* ^−*/*−^ mice, with significant movement of cells from the MZ (*Sema3F* ^+*/*+^ 15.09 ± 0.72%; *Sema3F* ^−*/*−^ 11.41 ± 0.52%, *p* < 0.03) into the CP (*Sema3F* ^+*/*+^ 35.66 ± 1.51%; *Sema3F* ^−*/*−^ 43.06 ± 1.05%, *p* < 0.001) (Supplementary Fig. 1d). Taken together, the observed changes in the number and laminar distribution of cortical interneurons suggest that there are alterations in their generation and/or migration in *Sema3A* ^−*/*−^ and *Sema3F* ^−*/*−^ mice.

### Deletion of semaphorins leads to alterations in cortical interneuron numbers in the developing striatum

**Fig. 3:**
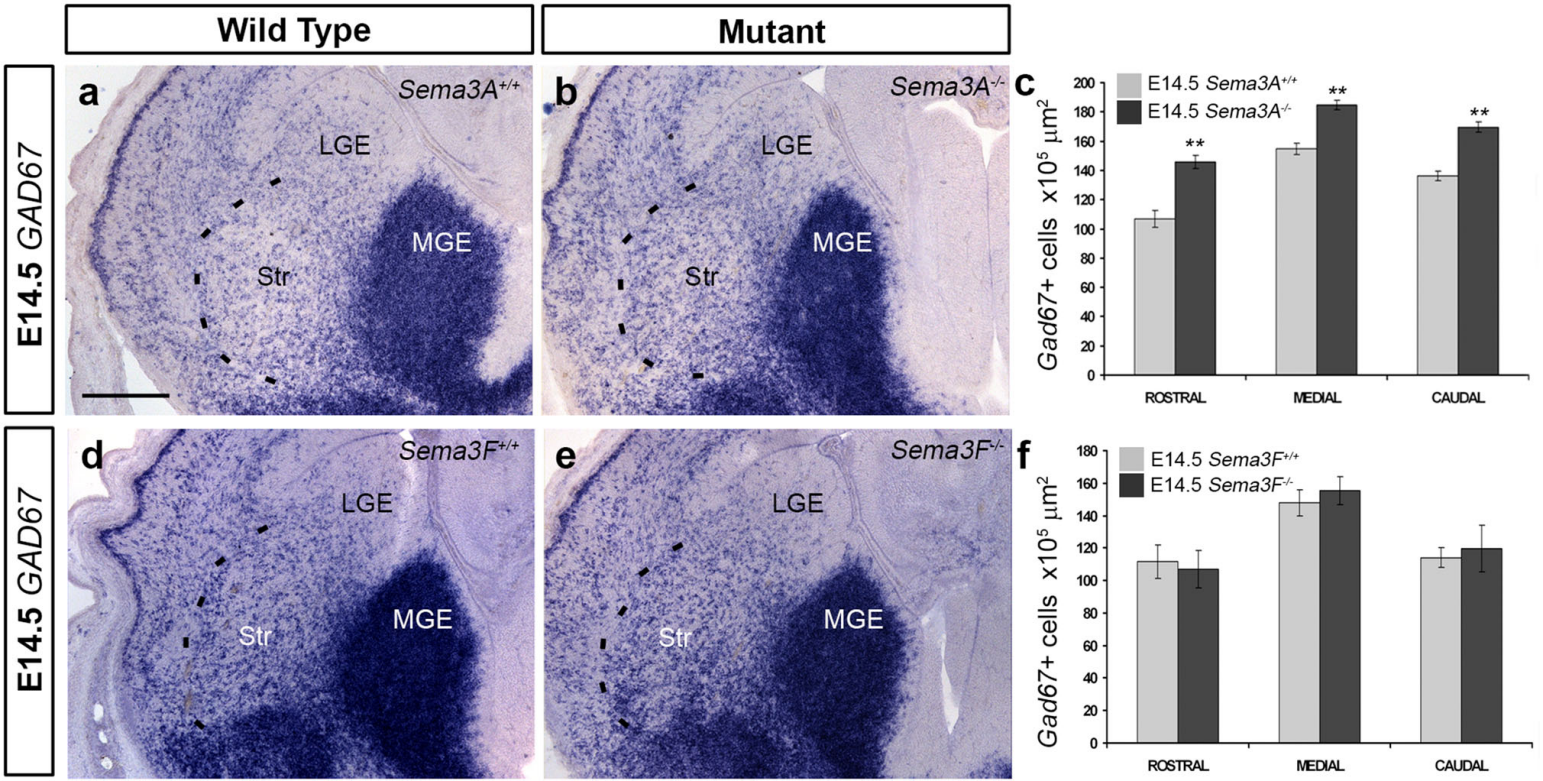
Altered number of neurons in the developing striatum of *Sema3A* ^−*/*−^ mice. *Images* of in situ hybridization for *GAD67* (**a**, **b**, **d**, **e**) in coronal brain sections from wild-type (**a**, **d**), *Sema3A* ^−*/*−^ (**b**) and *Sema3F* ^−*/*−^ (**e**) mice. Quantification of *GAD67* (**c**, **f**) cells in the striatum of semaphorin knockout animals showed increased number of labelled cells in *Sema3A* ^−*/*−^ (**c**), but not *Sema3F* ^−*/*−^ mice (**f**). *Scale bars* in **a** is 150 µm (Student’s *t* test, **p < 0.008). *Error bars* indicate SEM. *LGE* lateral ganglionic eminence, *MGE* medial ganglionic eminence, *Str* striatum

Previous studies have shown that altered expression of semaphorin receptors leads to aberrant migration of cortical interneurons through the striatum (Andrews et al. 2013; Hernandez-Miranda et al. 2011; Marin et al. 2001). To assess if the observed reduction in interneuron numbers in the cortices of semaphorin knockout mice was due to altered migration through this structure, we counted the number of GAD67+ cells throughout its rostral-caudal extent. We used GAD67 as a cortical interneuron marker in the striatum, as previous reports had shown that cortical interneurons contain high levels of GAD67, unlike striatal cells that preferentially express GAD65 (Feldblum et al. 1993; Greif et al. 1992; Mercugliano et al. 1992). At E14.5, we found a significant increase in the number of GAD67+ positive cells within the striatum of *Sema3A* ^−*/*−^ mice (middle level, *n* = 4, *Sema3A* ^+*/*+^ 154.36 ± 3.85; *n* = 4, *Sema3A* ^−*/*−^ 184.9 ± 3.14 × 10^5^ µm^2^, *p* < 0.0089) (Fig. *3*a–c), but not in *Sema3F* ^−*/*−^ animals (middle level, *n* = 4, *Sema3F* ^+*/*+^ 147.89 ± 7.99; *n* = 4, *Sema3F* ^−*/*−^ 155.47 ± 8.7 × 10^5^ µm^2^, *p* < 0.901) compared to control littermates (Fig. 3d–f). Similar findings were observed with the interneuron marker CB at the same age (data not shown). Interestingly, at E18.5, we observed a significant decrease in the number of GAD67+ cells in the striatum of *Sema3A* ^−*/*−^ mice, but only at middle levels (middle level, *n* = 4, *Sema3A* ^+*/*+^ 141.24 ± 5.13; *n* = 4, *Sema3A* ^−*/*−^ 118.39 ± 8.9 × 10^5^ µm^2^, *p* < 0.03), and no significant difference in *Sema3F* ^−*/*−^ mice (data not shown).

To assess whether this effect is specific to interneurons within the striatum, we immunostained coronal sections from *Sema3A* ^−*/*−^ mice and *Sema3A* ^+*/*+^ littermates for the transcription factor Forkhead box protein P2 (FOXP2), a marker of developing striatal projection neurons (Takahashi et al. 2003). Counts of labelled cells (*n* = 3 per age for each genotype) throughout the rostral-caudal extent showed a significant reduction in animals lacking the semaphorin at E14.5 (middle level: *Sema3A* ^+*/*+^ 529.55 ± 5.03, *Sema3A* ^−*/*−^ 451.12 ± 3.77 × 10^5^ µm^2^, *p* < 0.001), suggesting that deletion of Sema3A alters the number of striatal projection neurons, as well as cortical interneurons during development. A similar reduction in the number of striatal projection neurons was observed in *Sema3A* ^−*/*−^ mice using another marker, *ER81* (Stenman et al. 2003), but not in *Sema3F* ^−*/*−^ animals (data not shown). Our findings suggest that loss of Sema3A function in the ventral forebrain is affecting the generation and/or survival of multiple cell types in this region.

Before undertaking a study of proliferation in semaphorin null mice, we wanted to understand the reason for the increase in interneuron numbers in Sema3A null, but not in *Sema3F* ^−*/*−^ mice. We hypothesized that this may be due to differences in receptor expression levels, for example, there may be more interneurons expressing only Nrp1 (the preferred receptor for Sema3A) rather than Nrp2 (the nominal receptor for Sema3F). In terms of receptor expression at E14.5, approximately 85% of cultured MGE cells express Nrp1 (83.87 ± 4.665), 75% Nrp2 (74.66 ± 4.12%), and 70% (69.88 ± 4.05) express both receptors. This means that approximately 13% of cells only express Nrp1 receptor (83.87 Nrp1^+^—69.88% Nrp1^+^/Nrp2^+^cells). This is roughly the observed 16% increase in interneuron numbers entering the striatum in *Sema3A* ^−*/*−^ mice, suggesting that differences in receptor expression alone could explain the striatal phenotype in the absence of Sema3A.

### Reduced proliferation in the developing forebrain of *Sema3A*^−*/*−^ mice

**Fig. 4:**
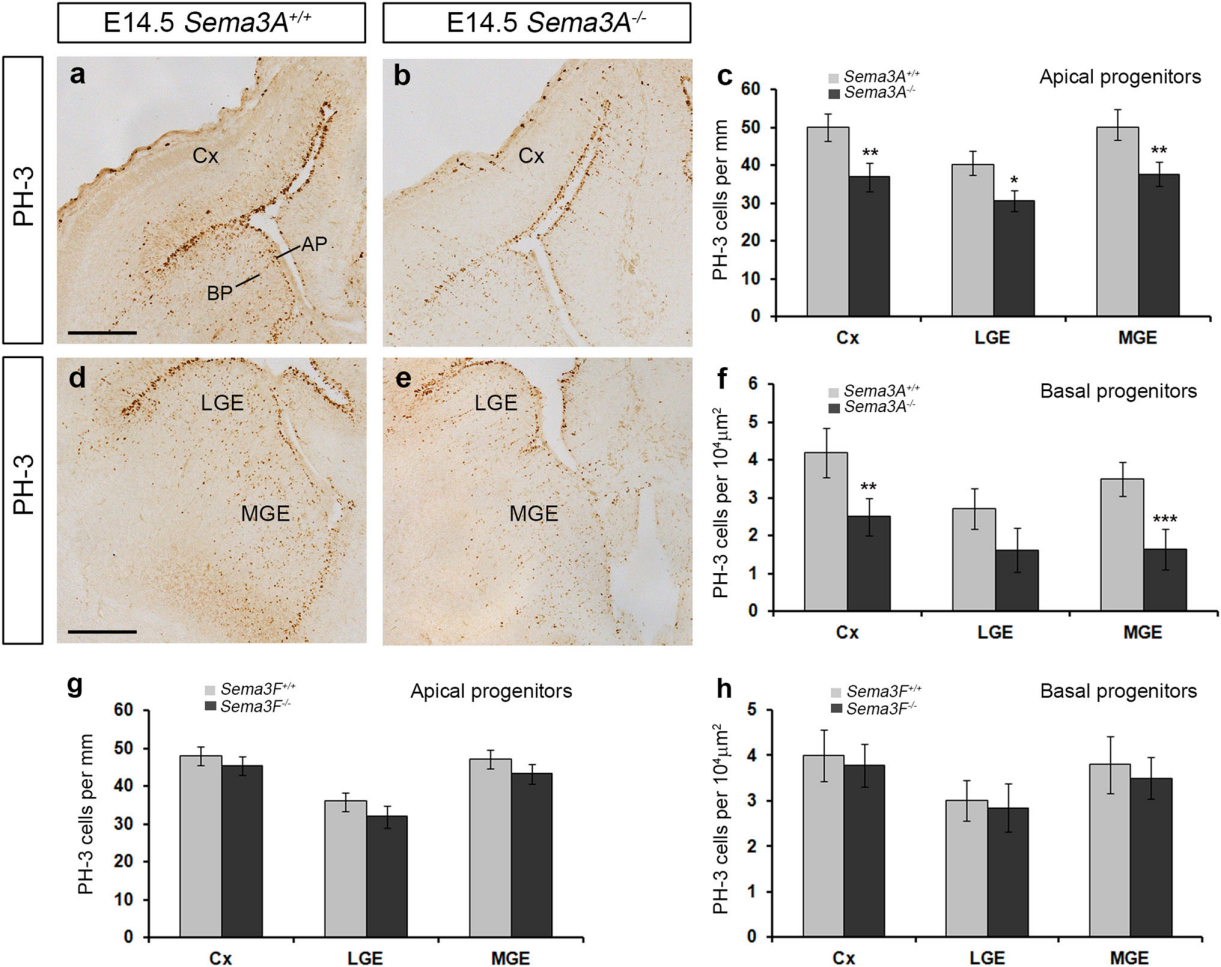
Reduced proliferation in *Sema3A* ^−*/*−^ mice. Coronal brain sections from *Sema3A* ^+*/*+^ (**a**, **d**) and *Sema3A* ^−*/*−^ (**b**, **e**) mice at E14.5 were immunostained for PH-3 (**a**, **b**, **d**, **e**). Quantification of PH-3^+^ apical (**c**, **g**) and basal progenitors (**f**, **h**) in the proliferative zones of *Sema3A* ^−*/*−^ animals showed reduced numbers when compared to *Sema3A* ^+*/*+^ littermates (**c**, **f**). No differences were observed in proliferation in *Sema3F* ^−*/*−^ mice (**g**, **h**). *Scale bar*s in **a**, **d** is 200 µm (Student’s *t* test, *p < 0.01, **p < 0.001). *Error bars* indicate SEM. *AP* apical progenitors, *BP* basal progenitors, *Cx* cortex, *MGE* medial ganglionic eminence, *LGE* lateral ganglionic eminence

We have recently shown that, similar to the present findings in the *Sema3A* ^−*/*−^ mice, loss of the semaphorin co-receptor PlexinA1 function leads to reduced proliferation, resulting in fewer cortical interneurons, as well as striatal interneurons and projection cells (Andrews et al. 2016). To determine whether the reduction in neuron numbers observed in *Sema3A* ^−*/*−^ mice was due to altered proliferation, we immunostained coronal sections from E14.5 *Sema3A* ^+*/*+^ and *Sema3A* ^−*/*−^ mice (*n* = 4 for both groups) for the mitotic marker PH-3. We found a significant decrease in the number of PH-3+ apical (*Sema3A* ^+*/*+^ 50.3 ± 4.82, *Sema3A* ^−*/*−^ 37.64 ± 3.28 per 100 µm, *p* < 0.002), as well as basal progenitors (middle level: *Sema3A* ^+*/*+^ 3.5 ± 0.45, *Sema3A* ^−*/*−^ 1.63 ± 0.53 per 10^4^ µm^2^, *p* < 0.0023) (Fig. 4a–f) in the MGE. A significant decrease was also found in the number of PH-3+ cells in the cortex (apical: *Sema3A* ^+*/*+^ 49.81 ± 3.59, *Sema3A* ^−*/*−^ 36.85 ± 3.7 100 µm, *p* < 0.002; basal: *Sema3A* ^+*/*+^ 4.2 ± 0.65, *Sema3A* ^−*/*−^ 2.5 ± 0.5 per 10^4^ µm^2^, *p* < 0.007) and in apical PH-3+ cells (*Sema3A* ^+*/*+^ 40.05 ± 3.79, *Sema3A* ^−*/*−^ 30.67 ± 2.76 100 µm, *p* < 0.01), but not basal progenitors (basal: *Sema3A* ^+*/*+^ 2.7 ± 0.54, *Sema3A* ^−*/*−^ 1.61 ± 0.58 per 10^4^ µm^2^, *p* < 0.1) in the LGE (Fig. 4a–e). No significant differences were noted in the number of PH-3 cells in *Sema3F* ^−*/*−^ mice compared to controls (Fig. 4g, h) (*n* = 4 each group).

To confirm these findings, E14.5 *Sema3A* ^−*/*−^ mice and their wild-type littermates (*n* = 4, *Sema3A* ^−*/*−^; *n* = 4, *Sema3A* ^+*/*+^) were pulse-labelled with BrdU, a thymidine analogue that becomes incorporated into DNA during S-phase of the cell cycle, for 1 h. We found a significant decrease in the number of BrdU+ cells in *Sema3A* ^−*/*−^ mutant mice compared to control littermates in the cortex (Cx: *Sema3A* ^+*/*+^ 82.51 ± 1.33, *Sema3A* ^−*/*−^ 71.08 ± 0.98 4 × 10^4^ µm^2^, *p* < 0.0385), LGE (LGE: *Sema3A* ^+*/*+^ 106.67 ± 2.57, *Sema3A* ^−*/*−^ 88.99 ± 1.05 4 × 10^4^ µm^2^, *p* < 0.017) and MGE (MGE: *Sema3A* ^+*/*+^ 92.93 ± 3.44, *Sema3A* ^−*/*−^ 74.53 ± 0.77 4 × 10^4^ µm^2^, *p* < 0.035). Taken together, these experiments suggest that there is a significant decrease in proliferation within the developing forebrain, particularly in the LGE and MGE, which would explain the observed reduction in striatal projection neurons and cortical interneurons in the developing forebrain of *Sema3A* ^−*/*−^ mice.

### Mice lacking semaphorin signalling through Nrp1 only partially phenocopy the cortical interneuron defects of *Sema3A*^−*/*−^ mice

Previous studies have shown, using Nrp1 dominant negative constructs and Nrp2 knockout mice, that loss of Nrp function perturbs the migration of cortical interneurons within the ventral telencephalon during early mid (E12.5–E15.5) phases of corticogenesis (Marin et al. 2001). Here, we wanted to analyse in greater detail the role of Nrp receptors in cortical interneuron development, and to assess which receptor is responsible for their altered migration in the striatum of *Sema3A* ^−*/*−^ mice. Because Nrp1 is considered the obligatory Sema3A receptor (Chedotal et al. 1998; Chen et al. 1998; He and Tessier-Lavigne 1997; Kolodkin and Ginty 1997), we anticipated that loss of semaphorin signalling through Nrp1 would phenocopy the cortical interneuron defect observed in *Sema3A* ^−*/*−^ mice. Thus, we analysed interneuron development in a Nrp1 mutant mouse strain that is deficient in semaphorin signalling through Nrp1, because it carries point mutations that prevent the interaction with the Sema domain (*Nrp1* ^*sema*−*/*−^, here in called *Nrp1* ^−*/*−^). We used these mice to circumvent the mid-gestation embryonic lethality of full *Nrp1* mutants caused by cardiovascular defects due to loss of VEGF signalling (Gu et al. 2003).

**Fig. 5:**
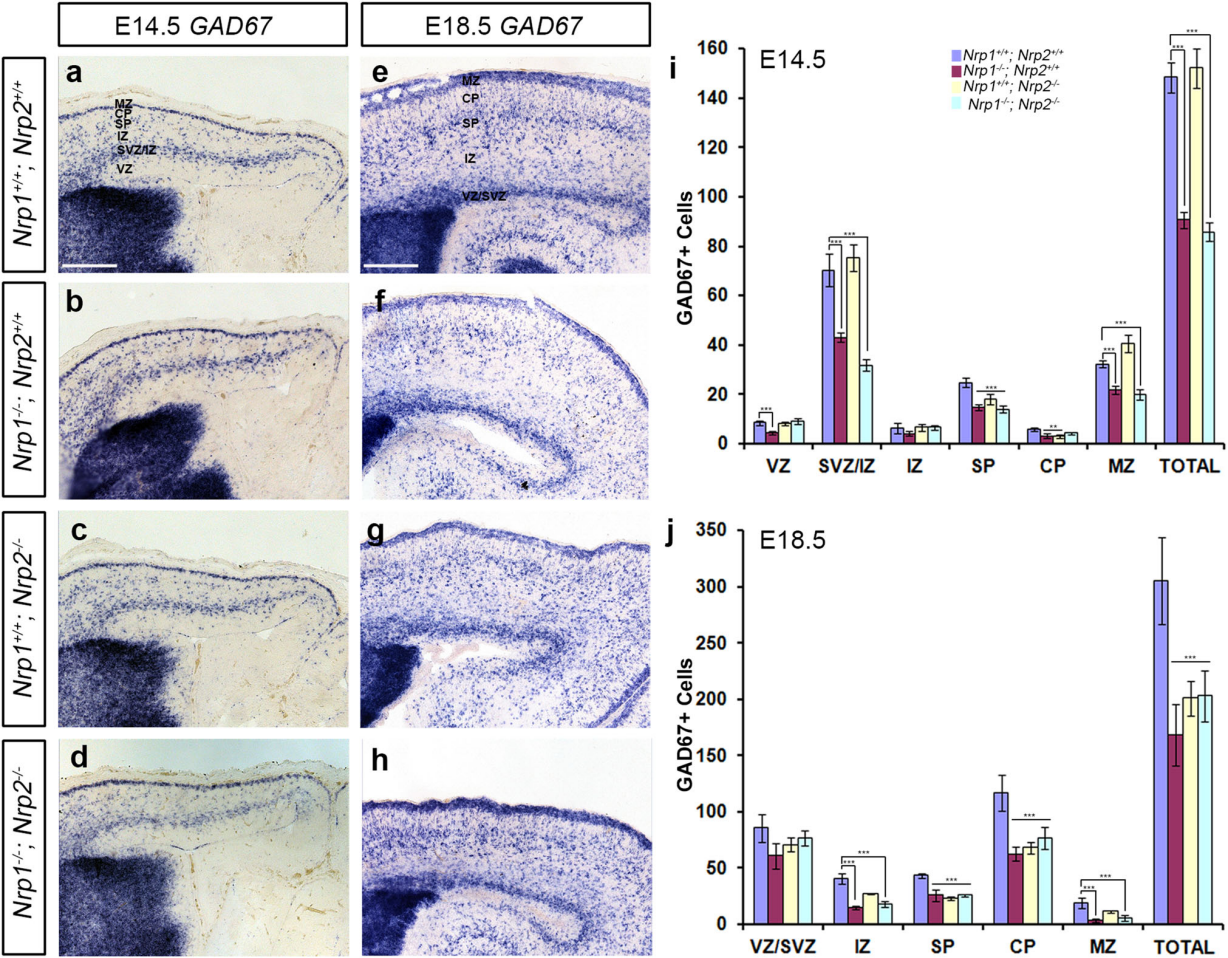
Altered number of GABAergic interneurons in the cerebral cortex of neuropilin knockout mice. Images of in situ hybridization for *GAD67* in coronal sections through the cortex of *Nrp1* ^+*/*+^; *Nrp2* ^+*/*+^ (**a**, **e**), *Nrp1* ^−*/*−^; *Nrp2* ^+*/*+^ (**b**, **f**), *Nrp1* ^+*/*+−^;*Nrp2* ^−*/*−^ (**c**, **g**), and *Nrp1* ^−*/*−^;*Nrp2* ^−*/*−^ at E14.5 (**a**–**d**) and E18.5 (**e**–**h**) mice. Analysis of the number and distribution of *GAD67* labelled cells in all layers of the cortex of neuropilin knockout mice at E14.5 (**i**) and E18.5 (**j**). Counts were made in the middle region along the rostro-caudal extent of the cortex. *Scale bar* in **a** and **e** is 150 µm. *Error bars* indicate SEM (one-way ANOVA, **p < 0.001, ***p < 0.0001). *CP* cortical plate, *IZ* intermediate zone, *MZ* marginal zone, *SP* subplate, *SVZ* subventricular zone, *VZ* ventricular zone

Using in situ hybridisation for *GAD67*, we assessed the number of interneurons within the developing cortex at middle (E14.5) and late (E18.5) stages in *Nrp1* ^−*/*−^ *;Nrp2* ^+/+^, *Nrp1* ^+*/*+^ *;Nrp2* ^−*/*−^, *Nrp1* ^−*/*−^ *;Nrp2* ^−*/*−^ double knockouts and control littermates (*n* = 3 all groups). We found a significant decrease in the total number of *GAD67*+ cells in the cortex in both *Nrp1* ^−*/*−^ *;Nrp2* ^+/+^ (*Nrp1* ^+*/*+^ *;Nrp2* ^+/+^ 148.33 ± 3.52; *Nrp1* ^−*/*−^ *;Nrp2* ^+/+^ 90.57 ± 3.09, *p* < 0.0158) and *Nrp1* ^−*/*−^ *;Nrp2* ^−*/*−^ double (*Nrp1* ^+*/*+^ *;Nrp2* ^+/+^ 148.33 ± 3.52; *Nrp1* ^−*/*−^ *;Nrp2* ^−*/*−^ 85.73 ± 3.7, *p* < 0.0007), but not in the *Nrp1* ^+*/*+^;*Nrp2* ^−*/*−^ (*Nrp1* ^+*/*+^ *;Nrp2* ^+*/*+^ 148.33 ± 6.12; *Nrp1* ^+*/*+^ *;Nrp2* ^−*/*−^ 152.08 ± 8.07, *p* < 0.628) compared to control littermates at E14.5 (Fig. 5a–d, i). When we looked at the relative number of GAD67+ cells in the cortex of these mice, we noticed that the laminar distribution of cells in both *Nrp1* ^−*/*−^ *;Nrp2* ^+/+^ and *Nrp1* ^−*/*−^ *;Nrp2* ^−*/*−^ double at E14.5 showed a similar pattern. Both showed a significant reduction of the cells in the SVZ/IZ (*Nrp1* ^+*/*+^ *;Nrp2* ^+/+^ 53.83 ± 0.64%; *Nrp1* ^−*/*−^ *;Nrp2* ^+/+^ 47.09 ± 1.04%, *p* < 0.0005; *Nrp1* ^−*/*−^ *;Nrp2* ^−/−^ 45.1 ± 3.21%, *p* < 0.0003), and a corresponding increase in the VZ (*Nrp1* ^+*/*+^ *;Nrp2* ^+/+^ 4.84 ± 0.16%; *Nrp1* ^−*/*−^ *;Nrp2* ^+/+^ 6.32 ± 0.15%, *p* < 0.02; *Nrp1* ^−*/*−^ *;Nrp2* ^−/−^ 6.44 ± 0.44%, *p* < 0.04) and MZ (*Nrp1* ^+*/*+^ *;Nrp2* ^+/+^ 22.88 ± 0.34%; *Nrp1* ^−*/*−^ *;Nrp2* ^+/+^ 25.1 ± 0.56%, *p* < 0.03; *Nrp1* ^−*/*−^ *;Nrp2* ^−/−^ 26.93 ± 0.66%, *p* < 0.003). Loss of Nrp2 function also led to redistribution of cells from the SVZ/IZ (*Nrp1* ^+*/*+^ *;Nrp2* ^+*/*+^ 53.83 ± 0.64%; *Nrp1* ^+*/*+^ *;Nrp2* ^−*/*−^ 46.27 ± 3.21%, *p* < 0.0008) into the IZ (*Nrp1* ^+*/*+^ *;Nrp2* ^+*/*+^ 3.12 ± 0.11%; *Nrp1* ^+*/*+^ *;Nrp2* ^−*/*−^ 7.48 ± 0.38%, *p* < 0.006) and MZ (*Nrp1* ^+*/*+^ *;Nrp2* ^+*/*+^22.88 ± 0.34%; *Nrp1* ^+*/*+^ *;Nrp2* ^−*/*−^ 26.14 ± 0.65%, *p* < 0.007) (Supplementary Fig. 2a).

Analysis at a later phase of corticogenesis (E18.5) similarly revealed significant differences in the total number of *GAD67*+ cells in the middle region of the cortex of *Nrp1* ^−*/*−^ *;Nrp2* ^+/+^, *Nrp1* ^−*/*−^ *;Nrp2* ^−*/*−^ double (*Nrp1* ^+*/*+^ *;Nrp2* ^+/+^ 305.45 ± 38.57; *Nrp1* ^−*/*−^ *;Nrp2* ^+/+^ 168.33 ± 26.88, *p* < 0.005; *Nrp1* ^−*/*−^ *;Nrp2* ^−*/*–^ 202.9 ± 22.35, *p* < 0.0008), but also in *Nrp1* ^+*/*+^ *;Nrp2* ^−*/*−^ mice (*Nrp1* ^+*/*+^ *;Nrp2* ^+/+^ 305.45 ± 38.57; *Nrp1* ^+*/*+^;*Nrp2* ^−*/*−^ 200.9 ± 815.36, *p* < 0.0006) compared to control littermates (Fig. 5e–h, j). Our *Nrp1* ^+*/*+^;*Nrp2* ^−*/*−^ E18.5 findings are also in agreement with previous reports of reduced interneuron numbers in the adult *Nrp2* ^−*/*−^ cortex (Gant et al. 2009). Alterations in the distribution of GAD67 cells were also observed at E18.5 following loss of neuropilin function. We noted a shift in the position of cells, with movement from the IZ (*Nrp1* ^+*/*+^ *;Nrp2* ^+*/*+^ 13.27 ± 1.38%; *Nrp1* ^−*/*−^ *;Nrp2* ^−*/*−^ 8.85 ± 1.2%, *p* < 0.03) and CP (*Nrp1* ^+*/*+^ *;Nrp2* ^+*/*+^ 6.22 ± 1.48%; *Nrp1* ^−*/*−^ *;Nrp2* ^−*/*−^ 2.78 ± 8.07%, *p* < 0.03) into the SVZ/IZ (*Nrp1* ^+*/*+^ *;Nrp2* ^+*/*+^ 27.98 ± 3.98%; *Nrp1* ^−*/*−^ *;Nrp2* ^−*/*−^ 37.82 ± 3.25%, *p* < 0.02) in *Nrp1* ^−*/*−^ *;Nrp2* ^−/−^ double knockouts, and to a lesser degree in *Nrp1* ^−*/*−^;*Nrp2* ^+/+^ mice, while loss of Nrp2 function led to a significant reduction in the number of cells in the SP only (*Nrp1* ^+*/*+^ *;Nrp2* ^+*/*+^ 28.45 ± 1.29; *Nrp1* ^+*/*+−^ *;Nrp2* ^−*/*−^ 25.38 ± 1.32, *p* < 0.04) (Supplementary Fig. 2b). Taken together, the results suggest alterations in the migration and/or generation of interneurons in Nrp receptor null mice, similar to those observed in Sema3A knockout animals.

### No change in cortical interneuron numbers in the developing striatum of Nrp knockout mice

**Fig. 6:**
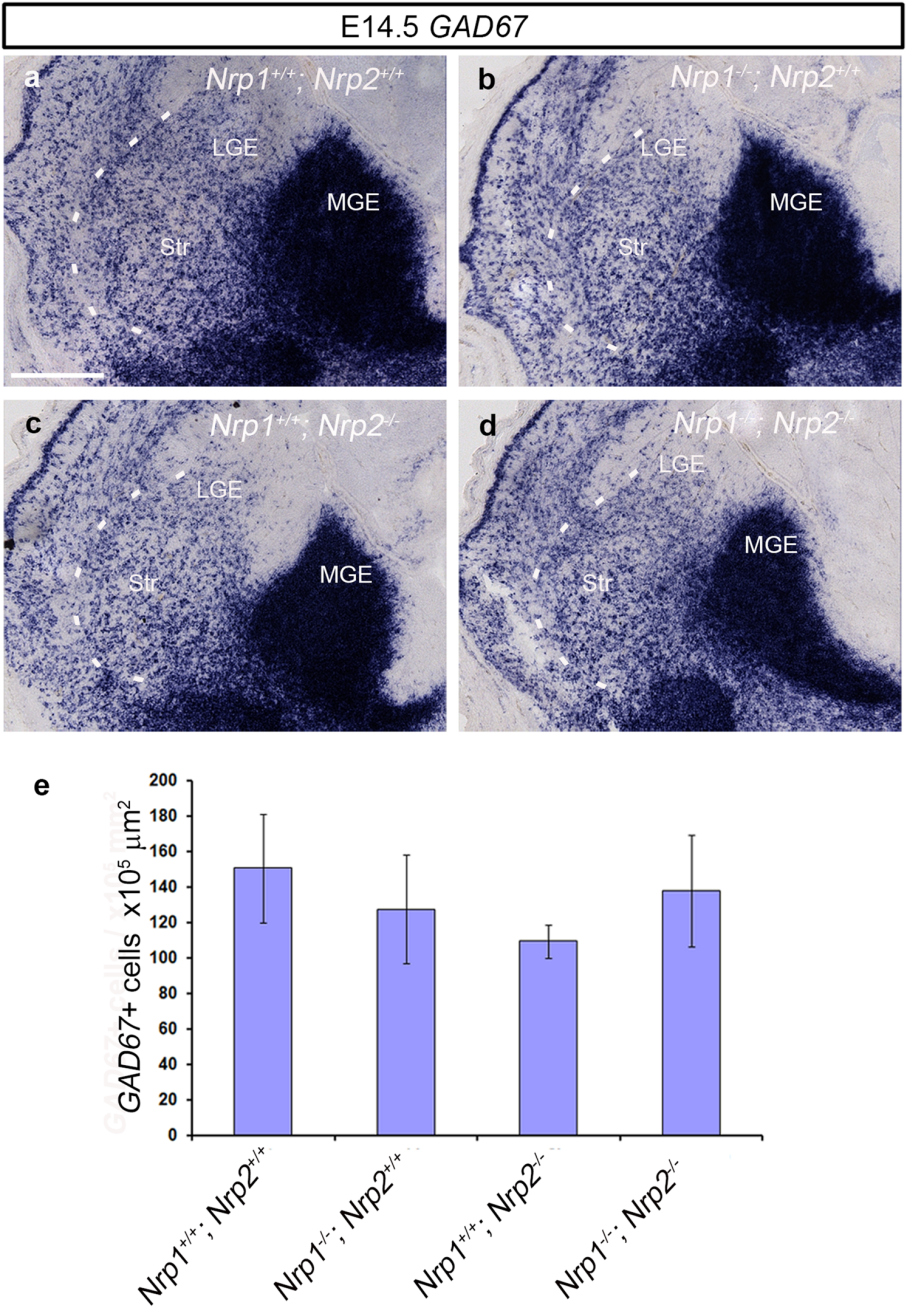
No change in the number of neurons in the developing striatum of neuropilin knockout mice. Images of in situ hybridization for *GAD67* (**a**–**d**) in coronal sections through the striatum of *Nrp1* ^+*/*+^; *Nrp2* ^+*/*+^ (**a**), *Nrp1* ^−*/*−^; *Nrp2* ^+*/*+^ (**b**), *Nrp1* ^+*/*+−^;*Nrp2* ^−*/*−^ (**c**) and *Nrp1* ^−*/*−^; *Nrp2* ^−*/*−^ (**d**) at E14.5. Quantification of *GAD67*+ cells in the striatum of neuropilin null mice showed no statistically significant differences compared to wild-type littermates (**e**). *Scale bar* in **a** is 200 µm. *Error bars* indicate SEM. *MGE* medial ganglionic eminence, *LGE* lateral ganglionic eminence, *Str* striatum

To establish whether the reduced number of interneurons in the cortex of neuropilin knockout mice is due to altered migration through the striatum, as previously proposed (Marin et al. 2001), we counted the number of *GAD67*+ cells throughout the rostral-caudal extent of this structure. In sections through the middle region, we found no significant changes in the number of *GAD67*+ cells at E14.5 (*Nrp1* ^+*/*+^ *;Nrp2* ^+/+^ 150.63 ± 30.55 × 10^5^ µm^2^; *Nrp1* ^−*/*−^ *;Nrp2* ^+/+^ 127.48 ± 30.58 × 10^5^ µm^2^, *p* < 0.615; *Nrp1* ^+*/*+^ *;Nrp2* ^−*/*−^ 109.53 ± 9.54 × 10^5^ µm^2^, *p* < 0.778; *Nrp1* ^−*/*−^ *;Nrp2* ^−*/*−^ 137.81 ± 31.43 × 10^5^ µm^2^, *p* < 0.268) (Fig. 6a–e) or at E18.5 (data not shown). Similar findings were observed with CB, suggesting that aberrant migration through the striatum is unlikely in the cause of the reduction of cortical interneurons in these mice. Indeed, the level of *GAD67* staining seen in the MGE appeared less in the neuropilin knockouts, particularly in the double *Nrp1* ^−*/*−^ *;Nrp2* ^−*/*−^ mutant, compared to control littermates (Fig. 6a, d), suggesting reduced proliferation or increased apoptosis are likely to be responsible for the phenotype.

### Reduced proliferation in the developing forebrain of *Nrp* knockout mice

**Fig. 7:**
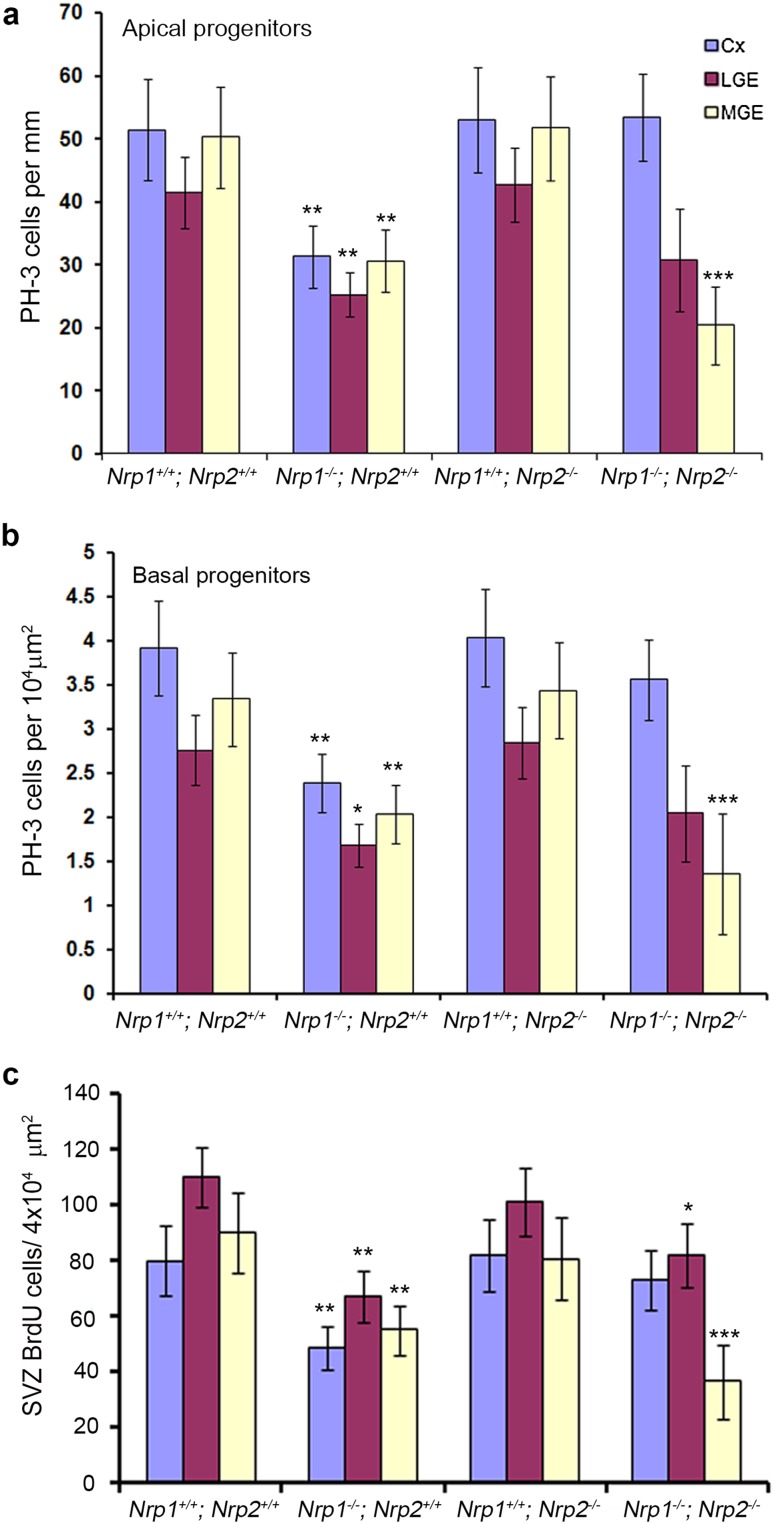
Reduced proliferation in neuropilin knockout mice. Quantification of PH-3^+^ (**a**, **b**) and BrdU^+^ (**c**) cells in the proliferative zones of neuropilin knockout animals showed reduced numbers when compared to control littermates (one-way ANOVA *p < 0.01, **p < 0.001, ***p < 0.0001). *Error bars* indicate SEM. *Cx* cortex, *MGE* medial ganglionic eminence, *LGE* lateral ganglionic eminence

The reduction in neurons observed in the cortex of Nrp knockout mice was similar to that observed in *Sema3A* ^−*/*−^ animals which showed a corresponding decrease in proliferation. To assess proliferation in these mice, we immunostained coronal sections from E14.5 Nrp single, double mutant mice and wild-type littermates (*n* = 3 for all groups) for the mitotic marker PH-3. At E14.5, we observed a significant decrease in the number of PH-3+ apical (*Nrp1* ^+*/*+^ *;Nrp2* ^+/+^ 50.25 ± 8.02 per mm; *Nrp1* ^−*/*−^;*Nrp2* ^+/+^ 30.65 ± 4.88 per mm, *p* < 0.001; *Nrp1* ^+*/*+^ *;Nrp2* ^−*/*−^ 51.76 ± 8.24 per mm, *p* < 0.628; *Nrp1* ^−*/*−^ *;Nrp2* ^−*/*−^ 20.4 ± 6.2 per mm, *p* < 0.0002), and basal progenitors in the MGE (*Nrp1* ^+*/*+^; *Nrp2* ^+/+^ 3.34 ± 0.53 per ×10^4^ µm^2^; *Nrp1* ^−*/*−^ *;Nrp2* ^+/+^ 2.04 ± 0.33 per ×10^4^ µm^2^, *p* < 0.001; *Nrp1* ^+*/*+^ *;Nrp2* ^−*/*−^ 3.44 ± 0.55 per ×10^4^ µm^2^, *p* < 0.628; *Nrp1* ^−*/*−^ *;Nrp2* ^−*/*−^ 1.36 ± 0.68 per ×10^4^ µm^2^, *p* < 0.0001) of *Nrp1* ^−*/*−^ *;Nrp2* ^+/+^ and *Nrp1* ^−*/*−^ *;Nrp2* ^−*/*−^ mice only (Fig. 7a, b). Similar reductions were observed in the LGE and cortex of *Nrp1* ^−*/*−^ *;Nrp2* ^+*/*+^ knockout mice only.

To confirm these findings, we pulse-labelled embryos at E14.5 with BrdU for 1 h, and similarly observed a significant decrease in the number of BrdU+ cells in *Nrp1* ^−*/*−^ *;Nrp2* ^+/+^ and *Nrp1* ^−*/*−^ *;Nrp2* ^−*/*−^ compared to control littermates in the MGE (*Nrp1* ^+*/*+^ *;Nrp2* ^+/+^ 89.95 ± 14.32 per ×10^4^ µm^2^; *Nrp1* ^−*/*−^ *;Nrp2* ^+/+^ 48.62 ± 8.74 per ×10^4^ µm^2^, *p* < 0.001; *Nrp1* ^+*/*+^ *;Nrp2* ^−*/*−^ 80.65 ± 14.75 per ×10^4^ µm^2^, *p* < 0.414; *Nrp1* ^−*/*−^ *;Nrp2* ^−*/*−^ 36.52 ± 13.26 per ×10^4^ µm^2^, *p* < 0.0004) (Fig. 7c). Taken together, these experiments suggest that there is a significant decrease in proliferation within the developing forebrain, particularly in the MGE, which would explain the observed reduction in cortical interneurons in *Nrp1* ^−*/*−^ *;Nrp2* ^+/+^ and *Nrp1* ^−*/*−^ *;Nrp2* ^−*/*−^ mice. These results are similar to those observed in the *Sema3A* ^−*/*−^ and the previously reported findings in *PlexinA1* ^−*/*−^ mice (Andrews et al. 2016), suggesting that Sema3A acting via Nrp1/PlexinA1 is involved in proliferation.

### Alterations in cytoskeleton and mitotic spindle orientation in MGE progenitors of *Sema3A*^−*/*−^ and *Nrp1*^−*/*−^ mice

Several lines of evidence have indicated a role for Sema3A in regulating proliferation in different cell types. First, the Sema3A-VEGF165 axis was shown to have a role in migration, apoptosis and proliferation in a neuroectoderm progenitor cell line (Bagnard et al. 2001) and, more recently, Sema3A has been specifically shown to regulate proliferation in activated satellite cells (Qahar et al. 2016). More pertinently, miR-30c and Sema3A have been reported to be involved in adult neurogenesis by regulating proliferation and differentiation of stem cells in the SVZ of mice (Sun et al. 2016). Interestingly, cerebrospinal fluid (CSF)-derived Sema3B was shown to orientate neuroepithelial cell divisions in the apico-basal axis within the developing neural tube via the same Nrp/PlexinA1 complexes (Arbeille et al. 2015).

**Fig. 8:**
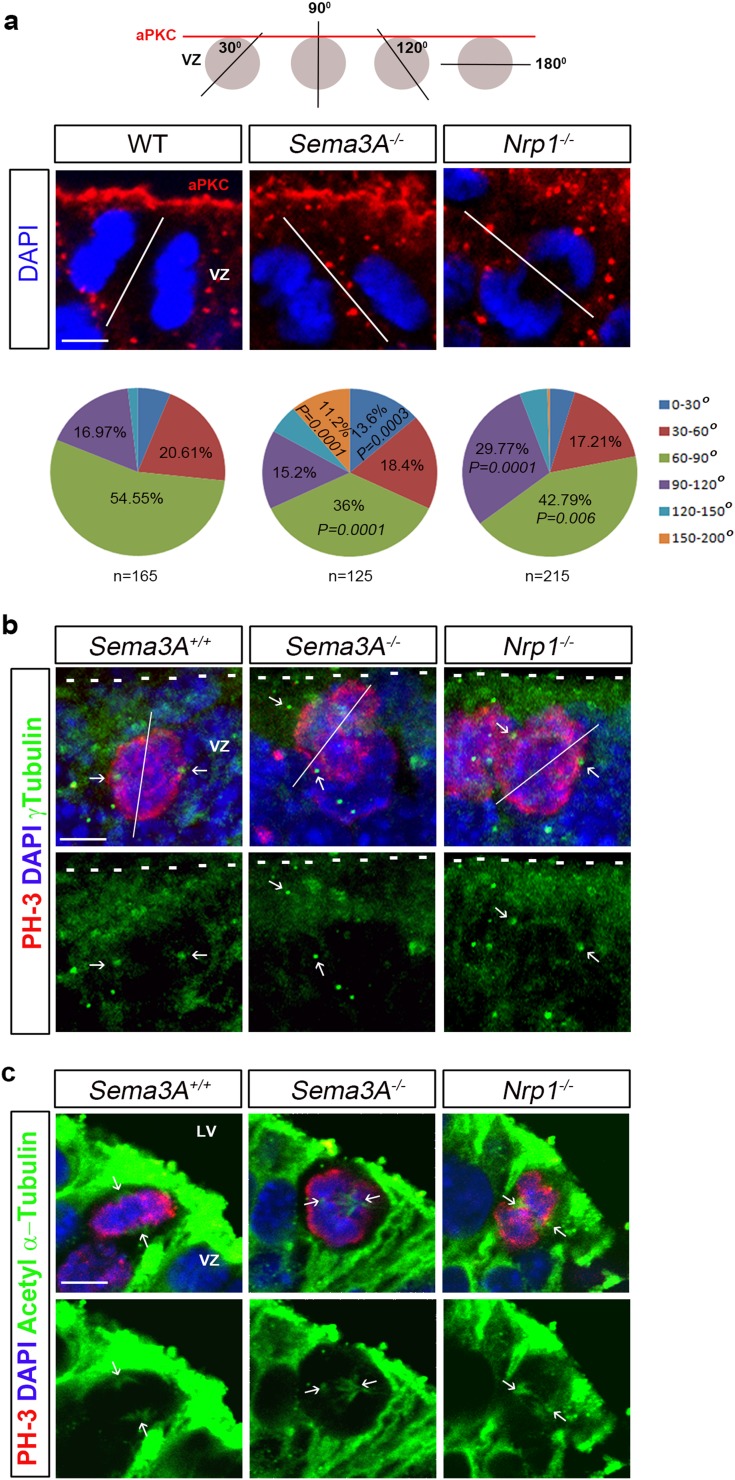
Altered cleavage plane in apical MGE progenitors in *Sema3A* ^−*/*−^ and *Nrp1* ^−*/*−^ mice. Coronal brain sections from wild-type, *Sema3A* ^−*/*−^ and *Nrp1* ^−*/*−^ mice at E12.5 were immunostained for aPKC (**a**), PH-3 (**b**, **c**), γ-Tubulin (**b**) and acetylated α-Tubulin (**c**); nuclei were counterstained with DAPI (**a**–**c**). Dotted lines denote position of VZ surface. Quantification of cleavage plane in MGE progenitors lining the VZ of *Sema3A* ^−*/*−^ and *Nrp1* ^−*/*−^ mice showed altered cleavage planes when compared to controls (**a**). Altered positions of centromeres (*arrows* in **b**) and microtubule assembly (*arrows* in **c**) are observed in *Sema3A* ^−*/*−^ and *Nrp1* ^−*/*−^ mice compared to controls. *Scale bars* in **a** and **b** is 5 µm; **c** is 10 µm. *LV* lateral ventricle, *VZ* ventricular zone

To determine whether Sema3A acting via Nrp1 is playing a similar role here, we stained coronal sections from the MGE of wild-type, *Sema3A* ^−*/*−^ and *Nrp1* ^−*/*−^ mice at E12.5 for the apical polarity protein aPKC. These experiments showed that the apico-basal polarity of MGE progenitor cells was not affected by the absence of Sema3A or Nrp1 (Fig. 8a). To assess whether neuroepithelial cell divisions were altered, we labelled nuclei of MGE progenitor cells with DAPI, and measured the angle of the cleavage plane in dividing cells at the ventricular surface during anaphase. We found that orientation of the cleavage plane was significantly different between wild-type and mutant animals, with fewer MGE progenitors in *Sema3A* ^−*/*−^ and *Nrp1* ^−*/*−^ mice having a cleavage angle of 60°–90° [WT 54.55% (90/165); *Sema3A* ^−*/*−^ 36% (45/125), *p* = 0.0001; *Nrp1* ^−*/*−^ 42.79% (92/215), *p* = 0.006]. We also observed significantly more dividing cells having a cleavage angle of 150°–200° in *Sema3A* ^−*/*−^ animals [WT 0% (0/165); *Sema3A* ^−*/*−^ 11.2% (14/125), *p* = 0.0001], and 90°–120° in *Nrp1* ^−*/*−^ mice [WT 16.97% (28/165); *Nrp1* ^−*/*−^ 29.76% (64/215), *p* = 0.0001] than wild-type controls (Fig. 8a). Taken together, these data suggest that Sema3A acting via Nrp1 has an effect on the apico-basal axis of cell divisions within the MGE, similar to that proposed for Sema3B.

To determine if alterations in the mitotic machinery was responsible for the altered cleavage planes in MGE progenitor cells, we immunostained coronal sections from E12.5 wild-type, *Sema3A* ^−*/*−^ and *Nrp1* ^−*/*−^ mice with γ- and acetylated-tubulin to visualise the positions of centrosomes and mitotic spindles, respectively. Our analysis showed that the positions of centrosomes (Fig. 8b) and the arrangements of the mitotic spindles (Fig. 8c) were altered in dividing cells in *Sema3A* ^−*/*−^ and *Nrp1* ^−*/*−^ mice compared to controls. These findings suggest that changes in the cleavage plane of dividing cells may underlie the observed reduced proliferation and, consequently, decrease in interneuron numbers in *Sema3A* ^−*/*−^ and *Nrp1* ^−*/*−^ mice.

## Discussion

Cortical GABAergic interneurons are generated in the ventral telencephalon and migrate tangentially to reach their final destinations in the neocortex and hippocampus (Metin et al. 2006). Because of their crucial role in cortical functions and the impact of their abnormal development on neurological disorders, the mechanisms underlying their generation and migration are of considerable interest.

We previously demonstrated that interneurons in Robo1 knockout (*Robo1* ^−*/*−^) mice have reduced expression of Nrp1 and PlexinA1, rendering them less responsive to the chemorepulsive effects of Sema3A and Sema3F in the developing striatum, resulting in their aberrant migration through this structure *en route* to the cortex (Andrews et al. 2013; Hernandez-Miranda et al. 2011). Our findings confirmed previous studies that used Nrp1-dominant negative constructs and *Nrp2* ^−*/*−^ knockout mice (Marin et al. 2001) and blocking experiments using anti-Nrp1 antibodies (Tamamaki et al. 2003a; Zimmer et al. 2010). Further, recent Nrp2 over-expression and knockdown studies have indicated that perturbation of the semaphorin–neuropilin signalling pathway alters the migratory pattern of interneurons within the GE (Kanatani et al. 2015). Interestingly, the study by Tamamaki et al. (2003a) also demonstrated that in vitro over-expression of class 3 semaphorins in the cortex could similarly alter cortical interneuron migration. Here, we wanted to expand on these studies and explore in greater detail the role(s) of semaphorin–neuropilin signalling in cortical interneuron development, using transgenic mice which lack either the Sema3A or Sema3F ligands and Nrp1 mice that do not bind these molecules, but are still able to maintain VEGF function, which is crucial for cortical interneuron development (Li et al. 2013).

We first used in situ hybridization to examine the mRNA expression of Sema3A/Sema3F throughout the developing mouse forebrain. These experiments revealed the presence of *Sema3A/Sema3F* in the striatum throughout development and, at E14.5, in cortical layers adjacent to the streams of migrating interneurons suggesting, in agreement with previous reports, that they may regulate their movement, both within the dorsal and ventral forebrain (Marin et al. 2001; Tamamaki et al. 2003a; Zimmer et al. 2010).

Using several specific markers, we found a significant decrease in the number of interneurons in the cortex of *Sema3A* ^−*/*−^ mice at E14.5 and E18.5. Interestingly, while loss of Sema3F function did not affect the number of interneurons, it did have a significant effect on their positioning within the cortex. Our expression analysis demonstrated strong expression of Sem3F in the CP at E18.5 and, in its absence, we observed movement of interneurons from the MZ and SP into the CP. Loss of either Nrp1 or Nrp2 function also had a small, but significant effect on interneuron positioning within the cortex, while loss of both neuropilins had a much more pronounced effect. Our findings indicate that Sema3F acting via neuropilins play an important role in interneuron positioning within the developing cortex.

The reduced number of interneurons in the cortex of *Sema3A* ^−*/*−^ mice may be due to the previously documented altered migration (Hernandez-Miranda et al. 2011; Marin et al. 2001; Tamamaki et al. 2003a). Indeed, we observed more cortical interneurons in the striatum of *Sema3A* ^−*/*−^ mice, and hypothesized that differences in Nrp receptor levels could potentially underlie this increase. In fact, the percentage of interneurons only expressing Nrp1 was similar to the proportional increase of interneurons in the developing striatum in Sema3A null mice. Interestingly, further analysis of the striatum and cortex revealed a decrease in the number of striatal projection neurons and cortical pyramidal cells as well as interneurons at later stages of development, suggesting that loss of Sema3A function could be affecting apoptosis or generation of different neural cell types in the developing forebrain.

Previous studies have highlighted the importance of programmed cell death in shaping the cortex throughout development (Haydar et al. 1999; Thomaidou et al. 1997), and prolonged exposure of Sema3A to neuronal progenitor cells and sensory neurons has been shown to induce apoptosis (Bagnard et al. 2001; Ben-Zvi et al. 2008). However, we failed to detect any changes in cell death in *Sema3A* ^−*/*−^ mice, indicating that apoptosis was unlikely the cause of the reduction of cortical interneurons in these animals. Thus, Sema3A must be having an effect on the generation of these cells. We have recently shown that loss of the semaphorin co-receptor PlexinA1 function leads to reduced proliferation, partly due to aberrant progenitor morphology and altered adhesiveness/attachment to the ventricular surface, resulting in fewer cortical and striatal interneurons as well as striatal projections cells (Andrews et al. 2016). These observations are very similar to those reported here for the *Sema3A* ^−*/*−^ and *Nrp1* ^−*/*−^ mice, indicating that Sema3A could be signalling via Nrp1–PlexinA1 during neurogenesis.

Cerebrospinal fluid-derived Sema3B acting via Nrp receptors was recently shown to orientate the apico-basal axis of neuroepithelial cell divisions in the developing mouse spinal cord (Arbeille et al. 2015). Relatively little is known about the molecular mechanisms that regulate the cleavage plane during division of MGE progenitors. Here, we show that progenitor cells in the VZ of the MGE in *Sema3A* ^−*/*−^ and *Nrp1* ^−*/*−^ mice exhibit differences in the angle of their cleavage plane. This could lead to changes in self-renewing cell divisions and progenitor cell fate, and may explain the observed reduction in cortical interneuron numbers in these mice, Interestingly, previous studies have shown that silencing the Ascl1-Rnd3 pathway in cortical progenitor cells alters the orientation of their cleavage plane and limits the divisions of progenitors, similar to that observed here in the MGE of *Sema3A* ^−*/*−^ and *Nrp1* ^−*/*−^ mice (Pacary et al. 2013). More recently, an interaction has been shown to exist between the cell-extrinsic Plexin signalling pathway and the cell-intrinsic Ascl1–Rnd3 pathway in cortical neurons (Azzarelli et al. 2014). Thus, it is possible that this particular pathway could be affected following loss of semaphorin–neuropilin–plexin signalling. However, the exact mechanism of how loss of Sema3A signalling via Nrp1 leads to altered cleavage plane and proliferation requires further study.

Whilst mice in which semaphorin signals through Nrp1 do phenocopy the reduced number of cortical interneurons observed in *Sema3A* ^−*/*−^ mice, they fail to copy the increased number of interneurons present in the developing striatum. These findings differ significantly from a previous study which used electroporation of Nrp1-dominant negative and GFP constructs into wild-type animals and GFP into *Nrp2* ^+*/*+^ and *Nrp2* ^−*/*−^ brain slices and reported a higher number of interneurons in the striatum (Marin et al. 2001). We can only suggest that differences in the experimental paradigms, methods of quantification or use of different genetic strains and background of the animals could all contribute to these contrasting results.

Our finding of differences in striatal phenotype between *Sema3A* ^−*/*−^ and *Nrp1* ^−*/*−^ mice indicates that other components must be involved in Sema3A signalling through this region. Previously, chondroitin-4-sulphate (CS) was shown to be co-expressed and bind to Sema3A in the striatal mantle, and both molecules can act in concert to repel cortical interneurons from this structure (Zimmer et al. 2010). This could possibly explain why loss of Nrp1 function does not recapitulate this phenotype. It is possible that both neuropilins may be required to mediate Sema3A signalling in guiding interneuron migration through the striatum. Earlier studies have shown that both Nrp1 and Nrp2 cooperate in Sema3A signalling events involved in regulating axon guidance and innervation in the sympathetic nervous system (Maden et al. 2012) and in gonadotropin-releasing hormone neuron migration (Cariboni et al. 2011). However, in our hands, loss of both neuropilin functions fails to rescue the striatal phenotype or to have an additive effect on the number of neurons or proliferating progenitor cells, compared to *Nrp1* ^−*/*−^ mice, confirming the redundant role for Nrp2 in striatal development. Interestingly, while we did not observe any changes in the number of neuronal progenitors or cortical interneurons at early stages of development in *Nrp2* ^−*/*−^ mice, we did find a marked decrease in interneuron numbers at E18.5. This finding is in agreement with previous studies which have shown reduced interneuron numbers and increased seizures in adult *Nrp2* ^−*/*−^ mice (Gant et al. 2009). Recently, in utero electroporation of Nrp2 knockdown constructs demonstrated that COUP-TFII/Nrp2 signalling acts as a molecular switch in determining the pathway and destination of migrating GABAergic neurons born in the preoptic area (Kanatani et al. 2015). Thus, further analysis is required to assess if cortical interneurons in our *Nrp2* ^−*/*−^ mice are misplaced, which could account for their altered number in the late stage of corticogenesis.

In summary, we have demonstrated that Sema3A is the key player in semaphorin signalling during interneuron migration and development. Absence of Sema3A or its receptor Nrp1 leads to a reduction in the size of progenitor pools in the developing MGE and, consequently, to reduced production of interneurons. We have also shown that Sema3A is the main ligand secreted from the striatum that is responsible for the repulsive cue preventing migrating interneurons from entering it. These results, together with our earlier findings, implicate Sema3A–Nrp1–PlexinA1 signalling events in the regulation of progenitor cell dynamics and interneuron migration in the developing ventral telencephalon.

## Electronic supplementary material

Below is the link to the electronic supplementary material.

Supplementary material 1 (TIFF 14914 kb) **Supplementary Fig.** **1**. Altered position of interneurons in semaphorin knockout mice. Histograms show the relative position of GAD67^+^ cells (**a-d**) in the cortex of *Sema3A* ^−*/*−^ (**a,c**) and *Sema3F* ^−*/*−^ (**b,d**) animals at E14.5 (**a,b**) and E18.5 (**c,d**) compared to control littermates. (Student’s *t* test, *P < 0.01, **P < 0.001). Error bars indicate SEM

Supplementary material 2 (TIFF 11940 kb) **Supplementary Fig.** **2**. Altered position of interneurons in neuropilin knockout mice. Histograms show the relative positions of GAD67^+^ cells (**a-d**) in the cortex of neuropilin knockout animals at E14.5 (**a**) and E18.5 (**b**) compared to control littermates. (one-way ANOVA, *P < 0.01, **P < 0.001, ***P < 0.0001). Error bars indicate SEM
