# Supplementary figures and images for: Semaphorin3A–neuropilin1 signalling is involved in the generation of cortical interneurons
Source: Brain Struct Funct. 2016 Nov 17;222(5):2217–33. doi: 10.1007/s00429-016-1337-3 (PMC5504245; doi:10.1007/s00429-016-1337-3)

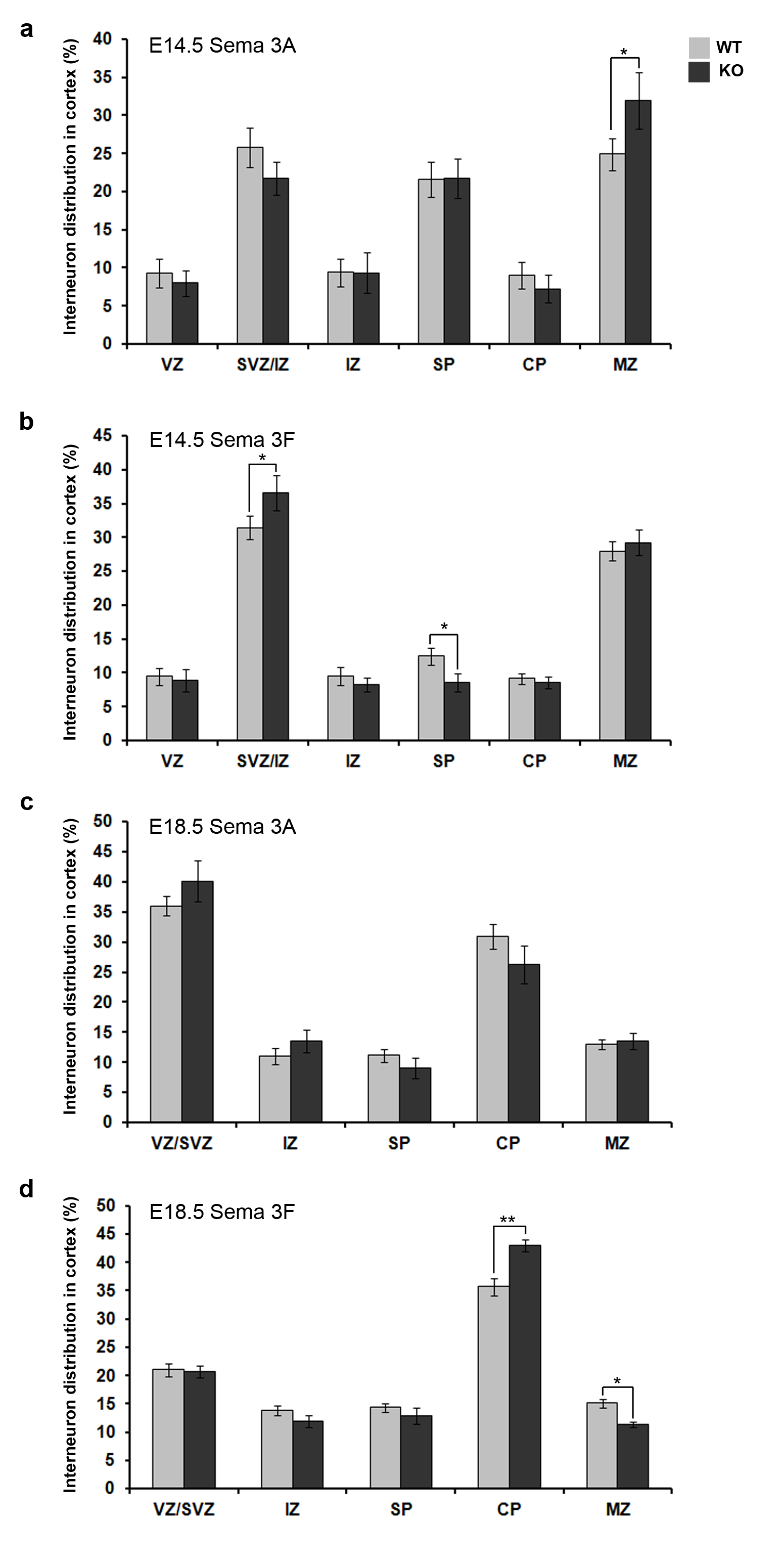

Supplement: Supplementary file 1 — Supplementary material 1 (TIFF 14914 kb) Supplementary Fig. 1. Altered position of interneurons in semaphorin knockout mice. Histograms show the relative position of GAD67+ cells (a-d) in the cortex of Sema3A −/− (a,c) and Sema3F −/− (b,d) animals at E14.5 (a,b) and E18.5 (c,d) compared to control littermates. (Student’s t test, *P < 0.01, **P < 0.001). Error bars indicate SEM [file 429_2016_1337_MOESM1_ESM.tif]

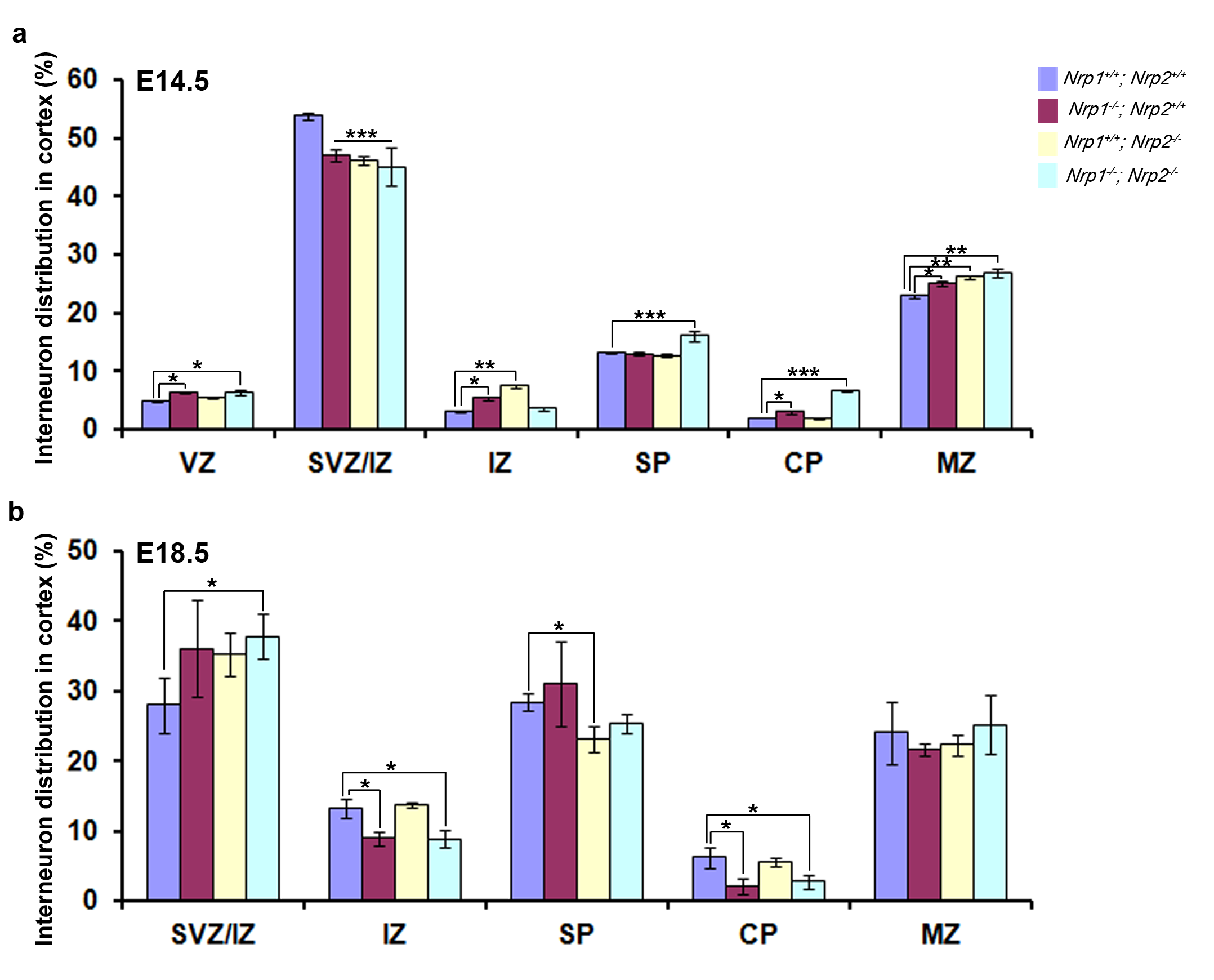

Supplement: Supplementary file 2 — Supplementary material 2 (TIFF 11940 kb) Supplementary Fig. 2. Altered position of interneurons in neuropilin knockout mice. Histograms show the relative positions of GAD67+ cells (a-d) in the cortex of neuropilin knockout animals at E14.5 (a) and E18.5 (b) compared to control littermates. (one-way ANOVA, *P < 0.01, **P < 0.001, ***P < 0.0001). Error bars indicate SEM [file 429_2016_1337_MOESM2_ESM.tif]
